# *Iocasia fonsfrigidae* NS-1 gen. nov., sp. nov., a Novel Deep-Sea Bacterium Possessing Diverse Carbohydrate Metabolic Pathways

**DOI:** 10.3389/fmicb.2021.725159

**Published:** 2021-11-24

**Authors:** Jing Zhang, Yuechao Zhang, Rui Liu, Ruining Cai, Fanghua Liu, Chaomin Sun

**Affiliations:** ^1^CAS Key Laboratory of Experimental Marine Biology and Center of Deep-Sea Research, Institute of Oceanology, Chinese Academy of Sciences, Qingdao, China; ^2^Laboratory for Marine Biology and Biotechnology, Qingdao National Laboratory for Marine Science and Technology, Qingdao, China; ^3^College of Earth Science, University of Chinese Academy of Sciences, Beijing, China; ^4^Center for Ocean Mega-Science, Chinese Academy of Sciences, Qingdao, China; ^5^School of Life Sciences, Hebei University, Baoding, China; ^6^Key Laboratory of Coastal Biology and Biological Resources Utilization, Yantai Institute of Coastal Zone Research, Chinese Academy of Sciences, Yantai, China

**Keywords:** fermentation, hydrogen, carbohydrates, cold seep, microcompartment

## Abstract

Resolving metabolisms of deep-sea microorganisms is crucial for understanding ocean energy cycling. Here, a strictly anaerobic, Gram-negative strain NS-1 was isolated from the deep-sea cold seep in the South China Sea. Phylogenetic analysis based on 16S rRNA gene sequence indicated that strain NS-1 was most closely related to the type strain *Halocella cellulosilytica* DSM 7362^T^ (with 92.52% similarity). A combination of phylogenetic, genomic, and physiological traits with strain NS-1, was proposed to be representative of a novel genus in the family Halanaerobiaceae, for which *Iocasia fonsfrigidae* NS-1 was named. It is noteworthy that *I. fonsfrigidae* NS-1 could metabolize multiple carbohydrates including xylan, alginate, starch, and lignin, and thereby produce diverse fermentation products such as hydrogen, lactate, butyrate, and ethanol. The expressions of the key genes responsible for carbohydrate degradation as well as the production of the above small molecular substrates when strain NS-1 cultured under different conditions, were further analyzed by transcriptomic methods. We thus predicted that part of the ecological role of *Iocasia* sp. is likely in the fermentation of products from the degradation of diverse carbohydrates to produce hydrogen as well as other small molecules, which are in turn utilized by other members of cold seep microbes.

## Introduction

Microbes inhabiting the deep sea represent a large portion of the biosphere, and resolving their ecology is crucial for understanding global ocean processes (Baker et al., 2021). In recent years, with more and more new species available in laboratory conditions, deep-sea sediments have been a prominent source of new lineages in the tree of life (Imachi et al., 2011, 2014; Hug et al., 2016; Nakahara et al., 2019; Lewis et al., 2020). Detailed investigations and comparisons of the metabolic potentials of these new lineages uncover key previously undescribed steps in element and nutrient cycling driven by previously uncultured microbes (Shah et al., 2017; Henson et al., 2018; Zhang et al., 2020). A recently isolated free-living representative of *Candidatus* Izemoplasma from the deep sea, which could degrade and utilize extracellular DNA (Zheng et al., 2021), was a good representative. Despite the global importance of these microorganisms, the majority of deep-sea microbial diversity remains uncultured and poorly characterized (Baker et al., 2021).

In the deep-sea ecosystem, heterotrophic microorganisms play an important role in the marine carbon cycle by breaking down polysaccharides and other organic biomass (Azam, 1998). The anaerobic degradation of organic matter involves complex microbial food chains, starting with the hydrolysis of macromolecular structures by extracellular enzymes and the formation of organic molecules small enough to be taken up by microorganisms (Arnosti, 2003; Liu et al., 2018). Microbial cells take advantage of these small organic molecules (such as sugars, amino acids, fatty acid, organic acids, and so on) through multistep fermentation processes with the production of a range of volatile fatty acids (VFAs), such as formate, acetate, propionate, and butyrate, together with H_2_ and CO_2_. Usually, these VFAs are electron donors that support the growth of diverse bacteria (Gittel et al., 2008; Zhang et al., 2019b; Choi et al., 2021).

Hydrothermal vents and cold seep zones are two special habitats in the deep sea. These habitats are always dark, and have extreme temperatures, and are rich in heavy metals and toxic substrates (Zhang et al., 2020; Zhou et al., 2020; Ma et al., 2021). The cold seep ecosystem is characterized by methane-rich migration, authigenic carbonate minerals, and chemosynthetic communities (Feng et al., 2018). Due to the distinct biogeochemistry dominated by fluid flow and hydrocarbon transport at the cold seep ecosystems, methanotrophs, hydrocarbon degraders, sulfate-reducing and sulfide-oxidizing bacteria are usually the key functional groups (Pachiadaki and Kormas, 2013). Taxonomically, the predominant bacterial communities in the cold seep are Proteobacteria, Firmicutes, Actinobacteria, Bacteroidetes, Planctomycetes, Nitrospirae, and Chloroflexi (Du et al., 2011; Jiao et al., 2015; Cui et al., 2019; Li et al., 2019). Notably, frequently reported fermentation microbiota are classified within the phyla of Bacteroidetes, Firmicutes, and Planctomycetes (Sun et al., 2016; Schwalm and Groisman, 2017; Zhang et al., 2017; Dedysh and Ivanova, 2019). The phylum Firmicutes is one of the predominant phyla in the cold seep, they include bacteria possessing diverse characteristics and have been abundantly detected in hydrate-bearing and crude oil deposit sites (Orcutt et al., 2010; Yamane et al., 2011). Clostridia are anaerobic Firmicutes producing a large array of metabolites by utilizing simple and complex carbohydrates and are often involved in the decomposition and recycling of organic matter (Penning and Conrad, 2006). The Clostridial bacteria are sometimes symbiotic with other organisms due to their production of alcohol (Ren et al., 2016), butanol (Lee et al., 2008), CO_2_, H_2_, and CO, through the fermentation of complex carbohydrates (Tracy et al., 2012), and several of the above products are important materials for the production of methane (Schink and Stams, 2013). Clostridia were found to be closely related to Methanogenesis and might be easy to understand (Sasaki et al., 2011). Undoubtedly, Clostridia are essential heterotrophic bacteria contributing to the whole bacterial community (Tracy et al., 2012). However, the studies of metabolic potentials conducted by deep-sea Clostridia lag well behind because of sampling and cultivation difficulties.

In the current study, a detailed description of the phenotypic traits, genomic data, and carbohydrate metabolisms was analyzed through biochemical and transcriptomic methods of the novel strain NS-1, which allows for the reconstruction of the metabolic potential and lifestyle of a novel Clostridial bacterium from the deep-sea. Based on the description of strain NS-1, we propose a novel species in a novel genus with strain NS-1 as its type strain, *Iocasia fonsfrigidae* gen. nov., sp. nov. Overall, this study widens understanding of diverse anaerobic categories and potential ecological roles in the deep-sea cold seep.

## Materials and Methods

### Isolation and Cultivation of Strain NS-1

Strain NS-1 was isolated from sedimentary samples (7–19 cm below the seabed) collected by ROV (remotely operated vehicle) *KEXUE* from the cold seep in the South China Sea (119°17′04.956′′E, 22°06′58.384′′N), at a depth of approximately 1,143 m in September of 2017. The temperature of the sampling site was 3.75°C, and the pH was 7.68 (Zhang et al., 2020), the total organic matter composition was about 0.6–0.9% (wt, %) (Li et al., 2016). The enrichment medium (EM) was modified from Zhang (2007) and contained 6.5 g PIPES, 25 g NaCl, 2.7 g MgSO_4_⋅7H_2_O, 4.3 g MgCl_2_⋅6H_2_O, 2.84 g Na_2_SO_4_, 0.25 g NH_4_Cl, 0.5 g KCl, 0.14 g CaCl_2_⋅2H_2_O, 0.14 g K_2_HPO_4_⋅3H_2_O, 0.002 g Fe(NH_4_)_2_(SO_4_)_2_⋅6H_2_O, 0.1 g yeast extract, 20 mM sodium lactate, 0.5 g cysteine-HCl and 0.001 g resazurin in 1,000 mL sea water filtered with 0.22μm filter membrane. The pH was adjusted to 6.5 with 2 M NaOH and the medium was boiled for several rounds to remove dissolved oxygen. Then 1 mL trace mineral element solution [containing 29 g Na_2_EDTA⋅2H_2_O, 3.26 g MnSO_4_⋅H_2_O, 1.8 g CoCl_2_⋅6H_2_O, 1 g ZnSO_4_⋅7H_2_O, 0.11 g NiSO_4_⋅6H_2_O, 0.1 g CuSO_4_⋅5H_2_O, 0.1 g H_3_BO_3_, 0.1 g KAl(SO_4_)_2_⋅12H_2_O, 0.1 g Na_2_MoO_4_⋅2H_2_O, 0.1 g Na_2_WO_4_⋅2H_2_O, 0.05 g Na_2_SeO_3_ in 1,000 mL Milli-Q water] and 10 mL vitamin solution (containing 2 mg biotin, 2 mg folic, 10 mg pyridoxine phosphate, 5 mg thiamine, 5 mg riboflavin, 5 mg niacin, 5 mg calcium pantothenate, 0.1 mg cobalamin, 5 mg para-aminobenzoic, and 5 mg thioctic acid in 1,000 mL Milli-Q water) were added to the autoclaved EM after being filtered with 0.22 μm filter membrane. Approximately 0.5 g sediment sample was added to 100 mL of EM and enriched for one month at 37°C. Unless otherwise specified, isolation and cultivation of microbes were performed by strict anaerobic procedures in an anaerobic chamber filled with 5% H_2_, 5%CO_2_, and 90% N_2_. To obtain pure cultures, enrichment was serially diluted tenfold in roll-tubes containing EM supplemented with 15 g/L agar (Hungate, 1969).

Purified strain NS-1 was cultured at 37°C in the PTYG medium containing 5 g peptone, 5 g tryptone, 10 g yeast extract, 4 g glucose, 5 g NaCl, 0.5 g cysteine-HCl, and 0.001 g resazurin in 1,000 mL artificial sea water (ASW). The ASW contained: 24.47 g NaCl, 3.917 g Na_2_SO_4_, 0.664 g KCl, 0.024 g SrCl, 4.981 g MgCl_2_⋅6H_2_O, 1.102 g CaCl_2_, 0.192 g NaHCO_3_, 0.026 g H_3_BO_4_ and 0.0039 g NaF per 1 l of Milli-Q water. The pH was adjusted to between 7.2 and 7.5 using 1 M NaOH.

### Analysis of Physiological Characteristics of Strain NS-1

Strain NS-1 was tested for the Gam reaction by using the Gram-staining method. Morphological characteristics of the cells were observed by transmission electron microscope (TEM) (HT7700, Hitachi, Japan) at 100.0 kV and zoom in 2,000. Determination of the temperature range for bacterial growth was tested using the PTYG medium over the range of 4–60°C (4, 16, 20, 28, 37, 45, and 60°C) for one week. To determine the need for NaCl for growth, NaCl-free artificial sea water was used to prepare the PTYG medium with different concentrations of NaCl. Briefly, 10 mL NaCl-free PTYG was respectively supplemented with 0, 12.5, 25, 50, 75, 100, 125, 150, 200, and 250 g/L NaCl, which was added into 15 mL Hungate tubes, and the culture was conducted at 37°C for one week. The pH range of growth was tested in triplicate from pH 3.36 to pH 9.36 (3.36, 4.04, 4.75, 5.32, 5.90, 6.48, 6.97, 7.6, 8.08, 8.45, 8.61, and 9.36), and 0.1 M citrate/sodium citrate buffer and 0.1M Tris-HCl/Tris-base buffer were used to prepare the PTYG medium, and the cultivation was performed for one week at 37°C. Susceptibility to antibiotics of strain NS-1 was tested in the PTYG medium supplemented with different antibiotics. The following antibiotics (Solarbio, Beijing) at an appropriate final concentration were tested: ampicillin (100 μg/mL), chloramphenicol (20 μg/mL), erythromycin (20 μg/mL), gentamicin (20 μg/mL), kanamycin (100 μg/mL), rifampicin (50 μg/mL), streptomycin (30 μg/mL), and vancomycin (30 μg/mL).

The anaerobic fermentation of carbon substrates by strain NS-1 was performed in triplicate by using the PTY medium (containing 5 g peptone, 5 g tryptone, 10 g yeast extract, 5 g NaCl, 0.5 g cysteine-HCl, and 0.001 g resazurin in 1,000 mL ASW) supplemented with different substrates at a final concentration of 0.5% (w/v). The use of different substrates was determined by comparison of the final biomass of strain NS-1. The following substrates were tested: L-arabinose, D-fructose, glucose, xylan, maltose, D-mannose, rhamnose, alginate, starch, sucrose, xylose, sodium ligninsulfonate (lignin), and galactose. PTY medium supplemented with different substrates were set as blank prior to incubation, and cultures without substrate in PTY medium were set as the control group.

Bacterial growth was monitored by measuring the amount of whole cell proteins (Begemann et al., 2012). Briefly, 0.5 mL culture was used to measure the whole cell proteins, cells were collected by centrifugation (8,000 rpm, 10 min, 4°C) washed twice with 10 mM PBS (pH = 7.4), and resuspend in 0.2 mL NaOH (0.1 M). Samples were boiled for 10 min and frozen in the refrigerator, repeated twice to break the cell completely. The major fermentation products of different substrates were determined after 48 h culture by High Performance Liquid Chromatography (HPLC) and gas chromatography (GC) as described previously (Zhang et al., 2019a). All samples were set up in triplicate and the final results were corrected by deducting the corresponding blank.

All statistically significant differences between groups were determined using Student’s *t*-test, and the Levene test was used to test whether the variance between the two groups was homogeneous. Statistical analysis was performed by SPSS Statistics 20 and *p* < 0.05 was considered as a significant difference. Congo red at a final concentration of 1 mg/mL was added to the PTY solid medium, supplemented with 1% carboxymethyl cellulose (CMC) to determine the CMC degradation ability of strain NS-1. For fatty acid composition assay, cell grown in PTYG medium were harvested after 48 h of incubation. The extracted fatty acids were analyzed by using an Agilent 6890 gas chromatograph (Agilent Technologies) and identified by using the Microbial Identification System operating manual (MIDI, 2008). Polar lipids of strain NS-1 were extracted using a chloroform/methanol system and analyzed by two-dimensional TLC (Thin Layer Chromatography) on silica gel 60 F254 aluminum-backed thin-layer plates (Merck). The plate dotted with the sample was subjected to two-dimensional development, with the first solvent system of chloroform: methanol: water (65: 25: 4, by Vol.) and the second solvent of chloroform: glacial acetic acid: methanol: water (80:18:12:5, by Vol.). Phosphomolybdate, ninhydrin, molybdenum blue, and 1-methylnaphthol were chosen as the chromogenic agents.

### Phylogenetic Analysis

Genomic DNA of strain NS-1 was extracted from the isolates, and PCR was performed to amplify the 16S rRNA gene sequence using primers 27F (5′-3′ AGAGTTTGATCMTGGCTCAG) and 1492R (5′-3′ TCAGGYTACCTTGTTACGACTT) as described previously (Wu et al., 2016). A phylogenetic tree based on the 16S rRNA gene for strain NS-1 and close phylogenetic relatives were created using MEGA X (Kumar et al., 2018). The tree was constructed by the maximum-likelihood method and the bootstrap support value 1,000.

### Genome Sequencing and Analysis

The genome of strain NS-1 was completely sequenced by Novogene Bioinformatic Technology Co. Ltd, Beijing, China using PacBio RSII. Pairwise genome comparison was performed with Average Nucleotide Identity (ANI) (Pritchard et al., 2016). Comparative genome analysis between strain NS-1 and *Halocella* sp. SP3-1 was carried out by using the MUMmer package (Delcher et al., 2003; Li and Ji, 2014). A detailed blast was run in Galaxy (Cock et al., 2015) to further compare the similarity of each coding sequence (CDS) in the two genomes.

### Transcriptional Profiling of Strain NS-1 Cultured With Different Carbohydrates

For transcriptomic profiling, strain NS-1 was grown in 10 mL PTY medium supplemented with CMC, rhamnose, lignin, and glucose at a final concentration of 0.5% (w/v), respectively. Cells cultured in the PTY medium were set as the control group. All samples were set up in triplicate. Cells were harvested after 24 h of incubation by centrifugation at 12,000 *g* for 10 min at 4°C. Three biologically repeated samples were mixed. Then the pellets were washed with 10 mM phosphate buffer solution (PBS pH 7.4), and total RNAs were extracted with Trizol reagent. Transcriptomic profiling was performed by Novogene Bioinformatics Technology Co. Ltd (Beijing, China) as described previously (Zhu et al., 2017). Raw data were filtered by removing reads containing adapter, reads containing poly-N, and low-quality reads. HTSeq v0.6.1 was used to count the read numbers mapped to each gene. The FPKM (Fragments Per Kilobase of transcript sequence per million of base pairs sequenced) of each gene was calculated based on the length of the gene and the read count mapped to this gene. Differential expression analysis of the two groups was performed using the DESeq R (1.18.0). The resulting *P*-values were adjusted using Benjamini and Hochberg’s approach for controlling the false discovery rate. Genes with an adjusted *P*-value < 0.05 found by DESeq were assigned as differentially expressed. To investigate the functional genes expressed under the treatment of different substrates, the relative expression of differentially expressed genes (DEGs) was further analyzed via heat-map (Deng et al., 2014) based on the log_2_(ΔFPKM-values). The ΔFPKM-values indicated the FPKM deviations between the substrate supplemented and the control group. The DEGs with different ΔFPKM-values, related to degradation of polysaccharide, production of ethanol, acetate, and hydrogen were chosen based on their reported function in the CAZY family, KEGG database, and previous studies ([Bibr B59][Bibr B28]).

## Results

### Isolation, Identification, and Characterization of the Novel Strain NS-1

We isolated the novel bacterial strains inhabiting the deep-sea cold seeps to gain insights into the diverse microbe-driven anaerobic metabolisms in the deep biosphere. As a result, a strictly anaerobic bacterial strain, designated NS-1, was isolated by several rounds of purification using the roll-tube method (Hungate, 1969). Analysis of the 16S rRNA sequence (1,444 bp) of strain NS-1 (accession No. MK418264.1) showed that it was a member of the family Halanaerobiaceae and possessed 92.52% sequence similarity (the highest score) with *Halocella cellulosilytica* DSM 7362^T^, a cellulose degradation bacterium isolated from the hypersaline (Simankova et al., 1993).

**FIGURE 1:**
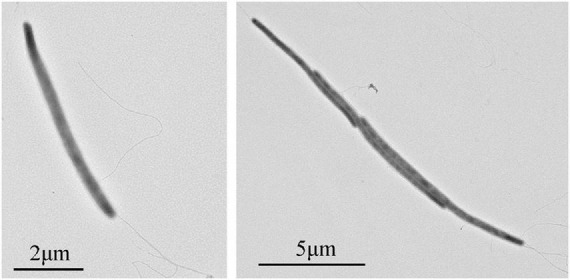
Transmission electron microscope observation of *I. fonsfrigidae* NS-1.

**FIGURE 2:**
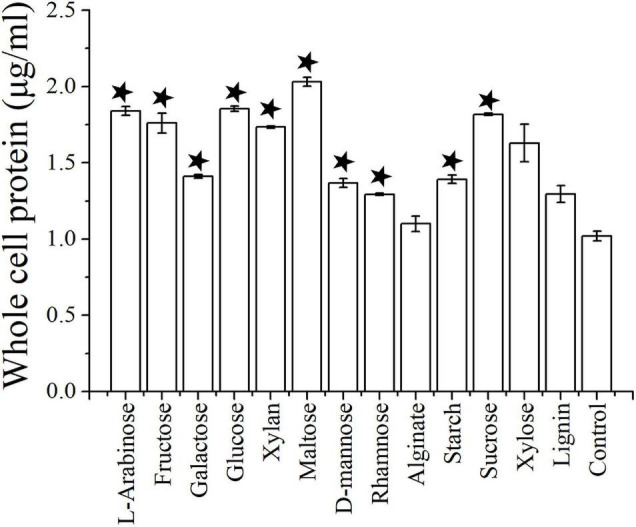
The growth assay of *I. fonsfrigidae* NS-1 under different carbohydrates by measuring the whole cellular protein. The error bars indicate the standard deviation (S.D.) from three different biological replicates. All statistically significant differences between groups were determined using Student’s *t*-test. The asterisk above each column indicates the growth of strain NS-1 is significantly (*p* < 0.05) promoted by the corresponding substrate.

Cells of strain NS-1 are long rods (0.2–0.3 μm in width, 6–10 μm in length), Gram-negative, and motile by flagella (Figure 1). The temperature and pH ranges for growth are 20–45°C and pH 6.5–8, and optimal growth occurs at 37°C and pH 7.0. The strain requires sodium chloride for growth at a concentration range of 12.5–150 g/L (optimal at 25–75 g/L). By measuring the amount of whole cellular protein of strain NS-1 growing under different conditions, it was clear that the growth of this bacterium was significantly increased with the supplement of respectively L-arabinose, fructose, galactose, glucose, xylan, maltose, D-mannose, rhamnose, starch, and sucrose in PTY medium (Figure 2). Due to the decolorization of the medium supplementing with lignin (Supplementary Figure 1), we deduced that the utilization of lignin occurred, though the growth of strain NS-1 was not significantly increased by the supplementation of lignin. Considering that strain NS-1 was closely related to the cellulose degrading bacterium, *H. cellulosilytica*, its cellulose degradation ability was tested. As expected, the CMC was observed to be consumed by strain NS-1 after three days of cultivation (Supplementary Figure 2A). Meanwhile, transparent zones appeared when strain NS-1 was cultured in the medium containing CMC supplementing with Congo red (Supplementary Figures 2B,C), indicating strain NS-1 could degrade CMC. The products of glucose fermentation are acetate, ethanol, lactate, butyrate, hydrogen, and carbon dioxide. The predominant membrane fatty acids comprise 15:0 anteiso (23.71%), 14:0 (17.93%), 15:0 iso (12.02%), and 16:0 (9.99%). The composition of polar lipids was diphosphatidylglycerol (DPG), phosphatidylglycerol (PG), unidentified phosphoglycolipids (PGL), and two unidentified glycolipids (GL) (Supplementary Figure 3). Strain NS-1 is sensitive to ampicillin (100 μg/mL), erythromycin (20 μg/mL), and rifampicin (50 μg/mL), while resistant to vancomycin (30 μg/mL), kanamycin (100 μg/mL), gentamicin (20 μg/mL), chloramphenicol (20 μg/mL), and streptomycin (30 μg/mL) (Table 1).

**TABLE 1:** Characteristics of strain NS-1 and other type strains of the genera in the family Halanaerobiaceae.

Strains	NS-1	*Halocella cellulosilytica* [1]	*Halothermothrix orenii* [2]	*Halarsenatibacter silvermanii* [3]	*Halanaerobium praevalens* [4]
Type strain	KCTC 15988 MCCC 1K04439	DSM 7362	DSM 9562	DSM 21684	DSM 2228
Cell size	0.2–0.3 × 6–10 μm	0.4–0.6 × 3.8–12 μm	0.4–0.6 × 10–20 μm	0.5 × 3 μm	0.9–1.1 × 2–2.6 μm
Morphology	Long rods	Rods	Rods	Curved rods	Rods
Motility	+*, flagella	+, peritrichous flagella	+, peritrichous flagella	+, paired subpolar flagella	–
Endospores	–*	–	–	–	–
Spheroplasts	–	+	NA	–	–
Gas vesicles	–	NA^#^	NA	–	NA
NaCl range	1.25–15%	5–20%	4–20%	20–35%	2–30%
NaCl optimum	2.5–7.5%	15%	10%	35%	13%
pH range	6.5–8	5.5–8.5	5.5–8.2	8.7–9.8	6.0–9.0
pH optimum	7	7	6.5–7.0	9.4	7.0–7.4
Temperature range	20–45°C	20–50°C	45–68°C	28–55°C	5–50°C
Temperature optimum	37°C	39°C	60°C	44°C	37°C
Carbohydrate utilized	+	+	+	–	+
Antibiotic sensitivity	Ampicillin (100 μg/mL), erythromycin (20 μg/mL) and rifampicin (50 μg/mL)	NA	NA	NA	NA
Antibiotic resistance	Vancomycin (30 μg/mL), kanamycin (100 μg/mL), gentamicin (20 μg/mL), chloramphenicol (20 μg/mL) and streptomycin (30 μg/mL)	NA	NA	NA	NA
Products of fermentation	Acetate, ethanol, lactate, butyrate, propionate H_2_, CO_2_	Acetate, ethanol, lactate, H_2_, CO_2_	Acetate, ethanol, H_2_, CO_2_	Not fermentative	Acetate, butyrate, propionate, H_2_, CO_2_
Major fatty acids	14:0 15:0 iso 15:0 anteiso	14:0 16:0 15:0 anteiso	14:0 15:0 iso 16:0	15:0 iso 18:0 17:0 iso 16:0	14:0 16:0 16:1
G + C content of DNA (mol%)	35.72	29	39.6	45.2	27
Sample source and site	Sediment, cold seep, South China Sea	Sediment, lake Sivash, Crimea	Sediment, hypersaline lake, Tunisia	Sediment, Searles Lake, CA, United States	Sediment, Great Salt Lake, UT, United States

* “+” indicates the strain has that capability; “–” indicates the strain lacks that capability.

*^#^NA indicates not available.*

*Related references cited in this table, (1) (Simankova et al., 1993), (2) (Cayol et al., 1994), (3) (Blum et al., 2009), and (4) (Zeikus et al., 1983).*

To further understand the characteristics of strain NS-1, its complete genome was sequenced. The size of the complete genome sequence of strain NS-1 is 3,926,493 bp with a G + C content of 35.72%. The genome harbors 3,700 coding sequences (CDSs), and 3,641 of them encode proteins. The genome of strain NS-1 contains a complete set of gene encoding enzymes related to glycolysis and pentose phosphate pathways, which are closely associated with carbohydrate metabolisms (Supplementary Figure 4). Correspondingly, quite a number of genes belonging to the CAZY family were identified in the genome (Supplementary Table 1). Among them the cellulase (*GM661_14245*) cellodextrinase (*GM661_08065*) and cellobiose phosphorylase (*GM661_04220*, *GM661_08070*, *GM661_08075*, *GM661_15060*, and *GM661_15065*) were responsible for the metabolism of cellulose, and the alpha-amylase (*GM661_00120*, *GM661_01725*, *GM661_02625*, and *GM661_03140*) was responsible for the degradation of starch. In comparison, genes related to the tricarboxylic acid (TCA) cycle are incomplete, indicating the key roles of carbohydrate metabolism toward energy production in strain NS-1.

**FIGURE 3:**
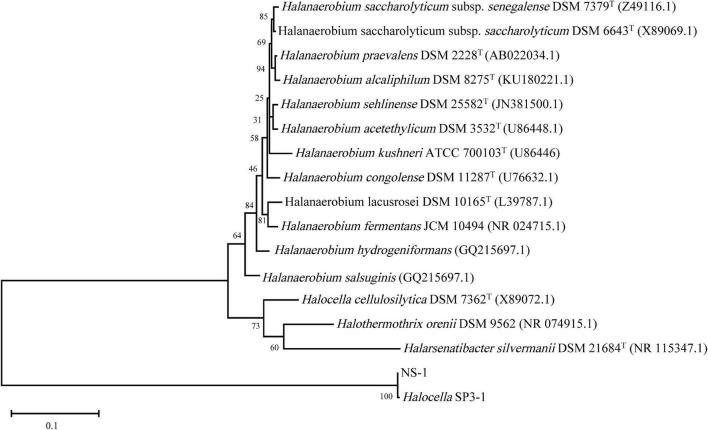
The consensus phylogenetic tree of strain NS-1 with other related relatives in the family Halanaerobiaceae obtained from the GenBank (accession numbers of different 16S rRNA sequences are indicated after the species name). The tree was constructed by the maximum-likelihood method and the bootstrap support values 1,000.

Phylogenetic analysis was conducted based on the 16S rRNA sequence of strain NS-1 and other species in the family Halanaerobiaceae (Figure 3). The low similarity between strain NS-1 and the genus *Halocella* indicated that this isolate represented a novel species of a potential novel genus as described before (Tindall et al., 2010; Yarza et al., 2014). Moreover, a phylogenomic analysis was performed based on the genomic information of strain NS-1 and other species in the family Halanaerobiaceae (Supplementary Figure 5). Therefore, we proposed strain NS-1 to be classified as the type strain of a novel species in a novel genus, for which the name *Iocasia fonsfrigidae* gen. nov., sp. nov. was proposed.

### Characterization of Anaerobic Metabolism of *Iocasia fonsfrigidae* NS-1

**FIGURE 4:**
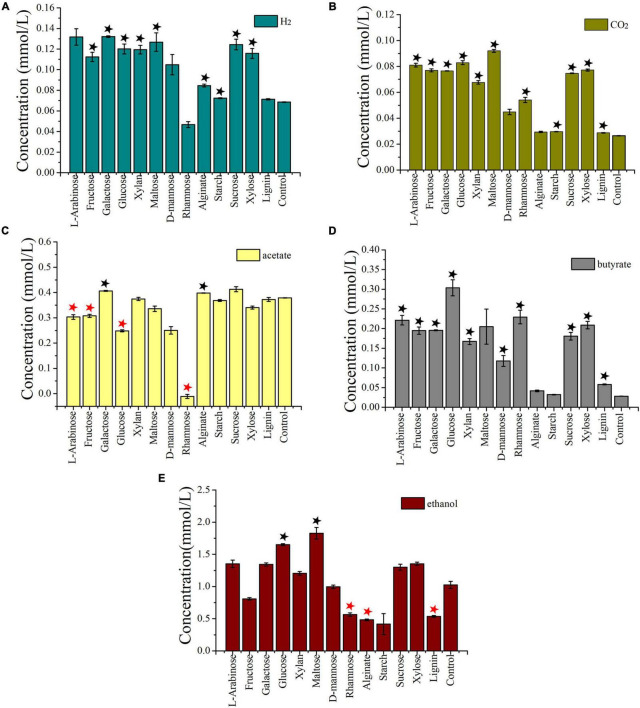
Detection and quantification of the production of H_2_ **(A)**, CO_2_, **(B)**, acetate **(C)**, butyrate **(D)**, and ethanol **(E)** by *I. fonsfrigidae* NS-1 incubated in the medium supplemented with different carbohydrates. The error bars indicate the standard deviation (S.D.) from three different biological replicates. All statistically significant differences between groups were determined using Student’s *t*-test. Asterisk indicates the production is significantly (*p* < 0.05) promoted (the black one) or inhibited (the red one).

As *I. fonsfrigidae* NS-1 was a strictly anaerobic bacterium utilizing various substrates as carbon sources (Table 1), we further particularly investigated its metabolic details toward different carbohydrates. To further explore the process of carbohydrate degradation by strain NS-1, fermentation products (including acetate, ethanol, butyrate, hydrogen, and carbon dioxide) from different substrates were determined. The production of hydrogen was significantly increased by the supplementation of fructose, L-galactose, glucose, xylan, D-maltose, alginate, starch, sucrose, or xylose (Figure 4A). The generation of carbon dioxide was significantly increased by almost all the tested carbohydrates (Figure 4B). The amount of produced acetate was significantly increased by supplementation of galactose or alginate, while markedly decreased by the supplement of L-arabinose, fructose, glucose, or rhamnose (Figure 4C). The production of butyrate was significantly increased by L-arabinose, fructose, galactose, glucose, xylan, D-mannose, rhamnose, sucrose, xylose, or lignin (Figure 4D). The yield of ethanol was significantly increased by the supplement of glucose or maltose, while it evidently decreased by supplementing rhamnose, alginate, or lignin (Figure 4E). Overall, *I. fonsfrigidae* NS-1 was shown to utilize diverse carbohydrates and thereby produce many small molecular substrates such as acetate, ethanol, butyrate, hydrogen, and carbon dioxide.

### Transcriptomic Profiling of Carbohydrates Degradation by Strain NS-1

According to the results of the substrate degradation assays performed above (Figures 3, 4), we were clear that strain NS-1 could use a variety of mono- and polysaccharides. To get further insights into the possible degradation process, a transcriptomic analysis of strain NS-1 incubated in the presence of different carbohydrates was performed. CMC, lignin, glucose, and rhamnose were chosen as representative substrates, according to their effects on the metabolism of strain NS-1. Among them, CMC and lignin are two kinds of difficult-to-degrade polysaccharides and utilization of rhamnose could dramatically decrease the production of hydrogen and acetate. Moreover, glucose is a commonly used monosaccharide for energy production and growth promotion of many bacteria. Based on the transcriptomic results, the global distribution of differentially expressed genes (DEGs) was indicated in the volcano plot (Supplementary Figure 6). The expressions of 130, 571, 476, and 592 genes were most up-regulated, while 130, 586, 441, and 694 genes were down-regulated in CMC, rhamnose, lignin, and glucose supplemented groups compared to the control group, respectively, indicating expressions of more genes were dramatically changed in the glucose supplemented group than those in other groups.

**FIGURE 5:**
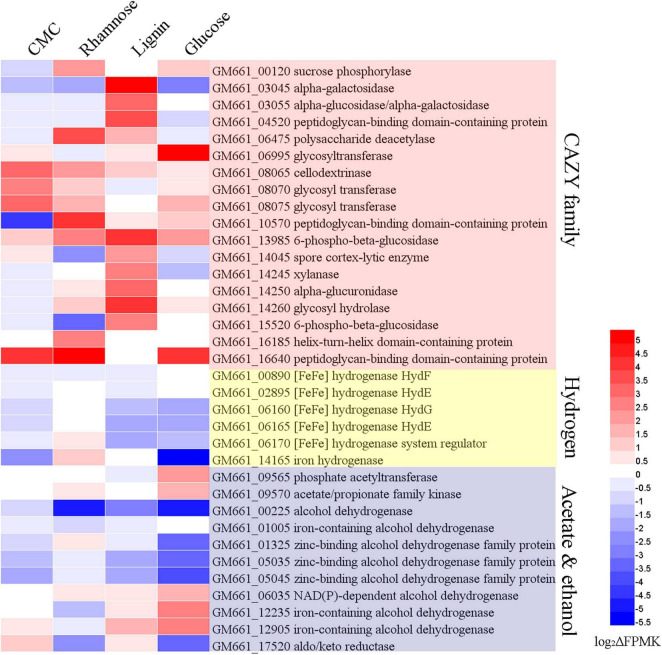
Transcriptomic analysis of *I. fonsfrigidae* NS-1 cultured in medium supplemented with different carbohydrates. Heat map showing differentially expressed genes encoding proteins associated with CAZY family, hydrogen, alcohol, and acetate production, which were respectively labeled with different colors. The color coding was related to the log_2_ΔFPMK values, the red colors represented a higher expression level and the blue ones represented a lower expression level. The heat map is generated by the Heml 1.0.3.3 software.

To investigate the functional genes expressed under the treatment of different substrates, the relative expression of DEGs was further analyzed via heat-map (Deng et al., 2014) based on the log_2_(ΔFPKM-values) (Supplementary Table 3). The ΔFPKM-values indicated the FPKM deviations between the substrate supplemented and the control group. By comparing the DEGs under different substrate supplement conditions, the most up-regulated or down-regulated genes that may be related to the degradation of carbohydrates, production of hydrogen, acetate, and ethanol were analyzed (Figure 5). Genes encoding CAZY family proteins related to carbohydrate degradation exhibited different expression levels according to different supplementing substrates. Of note, genes encoding cellodextrinase (*GM661_08065*) and glycosyltransferase (*GM661_08070* and *GM661_08075*) showed a relatively higher expression level in the CMC treatment group than other groups, which strongly indicated their potential function toward CMC degradation. These three genes belonged to the glycosyl hydrolase superfamily with putative carbohydrate binding domain (CBM), consistent with their potential cellulolytic function. The most up-regulated genes related to CMC degradation strongly indicated that CMC was an available substrate for strain NS-1.

As for the degradation of lignin, expressions of genes encoding alpha-galactosidase (*GM661_03045* and *GM661_03055*), peptidoglycan-binding domain-containing protein (*GM661_04520*), 6-phospho-beta-glucosidase (*GM661_13985*), spore cortex-lytic enzyme (*GM661_14045*), xylanase (*GM661_14245*), alpha-glucuronidase (*GM661_14250*), glycosyl hydrolase (*GM661_14260*), 6-phospho-beta-glucosidase (*GM661_15520*), and helix-turn-helix domain-containing protein (*GM661_16185*) were most up-regulated in the lignin supplement group than the other three groups. On the other hand, expressions of genes encoding sucrose phosphorylase (*GM661_00120*), polysaccharide deacetylase (*GM661_06475*), peptidoglycan-binding domain-containing protein (*GM661_10570*), helix-turn-helix domain-containing protein (*GM661_16185*), and peptidoglycan-binding domain-containing protein (*GM661_16640*) were markedly increased and these genes were considered to be key genes for the metabolism of rhamnose. Similarly, glycosyltransferase (*GM661_06995*) was the most up-regulated gene in the glucose treatment group and we thus propose that it is important in the glucose metabolism of strain NS-1.

Producing a large amount of hydrogen was an important metabolic feature of strain NS-1 in the presence of diverse carbohydrates (Figure 4A). Correspondingly, expressions of many genes related to hydrogen production were regulated under different treatments. Among them, expressions of genes encoding [FeFe] hydrogenase (*GM661_06160* and *GM661_06165*), [FeFe] hydrogenase system regulator (*GM661_06170*), and iron hydrogenase (*GM661_14165*) were most up- or down-regulated in the presence of different substrates, indicating the evident effects of different substrates on the production of hydrogen. Combining the production of hydrogen (Figure 4A) and the DEGs under the treatment of rhamnose and glucose (Figure 5), we found a negative relationship between the production of hydrogen and [FeFe]-hydrogenase related genes. The [FeFe]-hydrogenase was reported with particularly high turnover activities for both the release and oxidation of hydrogen (Land et al., 2020), and based on our results, we tended to believe that the main function of [FeFe]-hydrogenase in strain NS-1 was hydrogen oxidation.

Acetate was an important intermediate in a variety of metabolic processes, and its production was determined by indeterminate factors (Tracy et al., 2012). Up-regulated acetate/propionate family kinase (*GM661_09570*) and phosphate acetyltransferase (*GM661_09565*) in the glucose supplemented group, and up-regulated acetate/propionate family kinase (*GM661_09570*) in the rhamnose supplemented group might explain the decreased production of acetate in Figure 4C. As for the production of ethanol, we found two up-regulated iron-containing alcohol dehydrogenase (*GM661_12235* and *GM661_12905*) in the glucose supplemented group, which may be responsible for the increased production of ethanol.

**FIGURE 6:**
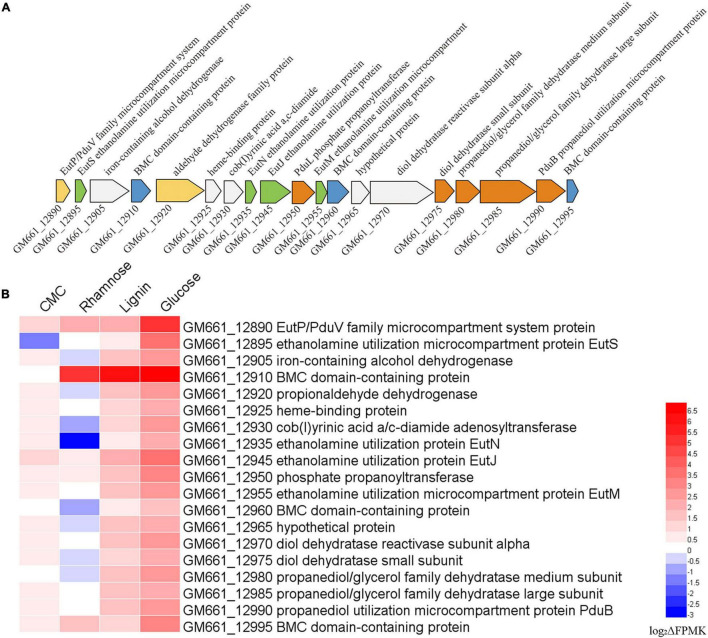
Transcriptomic analysis showing ethanolamine and propanediol metabolisms of *I. fonsfrigidae* NS-1 were affected by the presence of different carbohydrates and might be performed in microcompartment (BMC). **(A)** Gene cluster containing genes encoding different components responsible for ethanolamine and propanediol metabolism, as well as proteins constituting the bacterial BMC identified in the genome of *I. fonsfrigidae* NS-1. **(B)** Heat map showing the different expression levels of the genes belonging to the cluster shown in panel A when *I. fonsfrigidae* NS-1 incubated in the medium supplemented with different carbohydrates. The color coding was related to the log_2_ΔFPMK values, the red colors represented a higher expression level and the blue ones represented a lower expression level. The heat map is generated by the Heml 1.0.3.3 software.

It is noteworthy that an intact cluster containing genes encoding the different components responsible for ethanolamine and propanediol metabolism, as well as proteins constituting the bacterial microcompartment (BMC) were identified in the genome of strain NS-1 (Figure 6A). Moreover, the expression levels of most of these genes were up-regulated in the medium supplemented with CMC, lignin, or glucose (Figure 6B), strongly indicating ethanolamine and propanediol metabolisms were performed in BMC and affected by metabolized products from different carbohydrates.

### Comparative Genomic Analysis Between Strain NS-1 and *Halocella* sp. SP3-1

Recently, a genome sequence of *Halocella* sp. SP3-1 has been published (Heng et al., 2019) (accession number GCF_003953895.1), however, no further study was performed except the genome sequencing and polysaccharide hydrolase analysis. The 16S rRNA gene of strain NS-1 shared a high similarity of 99.58% with *Halocella* sp. SP3-1. The ANI between strain NS-1 and the *Halocella* sp. SP3-1 was 97.64%, which was higher than the accepted threshold (ANI value of 95–96%) for the same species (Richter and Rosselló-Móra, 2009). The ANI values between strain NS-1 and other types of strains in the family Halanaerobiaceae were lower than the accepted threshold (Supplementary Table 4). The high similarity of the 16S rRNA gene and the ANI value strongly indicated that *Halocella* sp. SP3-1 and strain NS-1 belonged to the same species. Logically, full polyphasic taxonomic description and detailed physiological characteristics were necessary before claiming a novel microorganism (Imachi et al., 2014; Oren, 2014), thus *Halocella* sp. SP3-1 needs to be recognized without valid standing in nomenclature. In the current study, we proposed that *Halocella* sp. SP3-1 belongs to the new species *Iocasia fonsfrigidae*. Considering that these two strains were isolated from different environments, a genomic comparison between these two strains was performed to identify the potential differences between these two strains. Although the genomic alignment analysis between strain NS-1 and *Halocella* sp. SP3-1 showed a very high similarity (Supplementary Figure 7), many proteins specifically existed in the genome of strain NS-1 but are absent in *Halocella* sp. SP3-1 were identified by Galaxy (Cock et al., 2015), such as proteins related to ABC transporters, PTS sugar transporters, type II secretion systems, type I-B and type III-B CRISPR associated proteins, proteins involved in carbohydrate metabolisms, and clusters of proteins related to ethanolamine and propanediol metabolism and BMC (Supplementary Tables 1, 2).

### Description of *Iocasia* gen. nov.

*Iocasia* (I.o.c.a.s’ia L.fem. n. *Iocasia* arbitrary name formed from the acronym of the Institute of Oceanology, Chinese Academy of Sciences). *Iocasia* gen. nov. belong to the family Halanaerobiaceae. Cells are long rods, with 0.2–0.3 × 6–10 μm. Cells were strictly anaerobic and moderately halophilic. Cells are Gram-negative and motile by flagella. The G + C content was between 35.1 and 35.72%. The type species is *I. fonsfrigidae* which was isolated from cold seep sediment from the South China Sea (Supplementary Table 5).

### Description of *Iocasia fonsfrigidae* sp. nov.

*Iocasia fonsfrigidae* (fo.ns.fri.gi.da’e. L. m. n. fons seep; L. fem. frigida cold: N. L. gen. n. fonsfrigidae of the cold seep). Cells are long rods (0.2–0.3 μm in width, 6–10 μm in length), Gram-negative and motile by flagella. The temperature and pH ranges for growth are 16–45°C and pH 6.5–8, and optimal growth occurs at 37°C and pH 7.0. The strain requires sodium chloride for growth at a concentration range of 12.5–150 g/L (optimal at 25–75 g/L). Substrates used as carbon sources are arabinose, D-fructose, galactose, glucose, xylose, maltose, D-mannose, rhamnose, alginate, sucrose, xylan, starch, lignin, and carboxymethylcellulose (CMC). The products of glucose fermentation are acetate, ethanol, lactate, butyrate, hydrogen, and carbon dioxide. The predominant membrane fatty acids comprise 15:0 anteiso (23.71%), 14:0 (17.93%), 15:0 iso (12.02%), and 16:0 (9.99%). The composition of polar lipids was diphosphatidylglycerol (DPG), phosphatidylglycerol (PG), unidentified phosphoglycolipids (PGL), and two unidentified glycolipids (GL). Strain NS-1 is sensitive to ampicillin, erythromycin, and rifampicin, while resistant to vancomycin, kanamycin, gentamicin, chloramphenicol, and streptomycin. The type strain NS-1^T^ (KCTC 15988^T^ = MCCC 1K04439^T^) was isolated from deep-sea sediment of cold seep, China, and deposited in Marine Culture Collection of China and Korea Collection for Type Cultures.

## Discussion

The estimated total microbial abundance was about 2.9 × 10^29^ cells in the global subseafloor sediment, and the living biomass was 10–45% lower than this estimate (Kallmeyer et al., 2012). These microbial communities process both organic and inorganic carbon and contribute to the cycling of nutrients such as sulfur, nitrogen, and iron (Parkes et al., 2014). To explore the potential novel metabolisms and ecological roles of microorganisms in the subseafloor, pure isolates are necessary. However, due to the sampling and cultivation limitations, most of the microorganisms in the subseafloor are uncultured (Martiny, 2019), especially those in deep-sea special environments such as hydrothermal vents and cold seep areas. In the present study, a novel genus and species *Iocasia fonsfrigidae* NS-1 belonging to the family Halanaerobiaceae was isolated from the deep-sea cold seep, and its physiological, phylogenetics, genomic, and metabolic traits were investigated in detail.

The family Halanaerobiaceae belong to the class Clostridia in the phylum Firmicutes was created in 1995 (Rainey et al., 1995). It contained 4 genera including *Halanaerobium* (Zeikus et al., 1983), *Halocella* (Simankova et al., 1993), *Halothermothrix* (Cayol et al., 1994), and *Halarsenatibacter* (Oremland et al., 2005; Blum et al., 2009). Most isolated species in Halanaerobiaceae belong to the genus *Halanaerobium* (Oren, 2014). All previous Halanaerobiaceae species were isolated from saline, sediments of salt or hypersaline lakes, offshore fields, petroleum reservoirs, and fermented puffer fish ovaries (Oren, 2014). *I. fonsfrigidae* NS-1 represents the first member of Halanaerobiaceae isolated from the sediment of a deep-sea cold seep. The natural habitats for these known Halanaerobiaceae populations reflect their capacity to tolerate a broad range of salt concentrations. Compared with other members of Halanaerobiaceae, *I. fonsfrigidae* NS-1 showed a relatively lower tolerance to the presented salt content in the medium, which could be a long-term adaptation to the original ecological habitat. The isolation of *I. fonsfrigidae* NS-1 from the cold seep strongly indicates that there exists a wide range of living habitats of Halanaerobiaceae, and their potential metabolic differences need further study in the future.

The ability to metabolize diverse carbohydrates by *I. fonsfrigidae* NS-1 is consistent with other members in the family Halanaerobiaceae (Blum et al., 2009). Strain NS-1 was shown to degrade cellulose (Supplementary Figure 2), and most marine cellulolytic bacteria belong to the genera *Bacillus* and *Clostridium* (Castro da Silva and de Souza Lima, 2017). The discovery of cellulolytic function in the genus *Iocasia* provides evidence for the microorganisms that possess the capability to decompose high-molecular-weight organic matter in the anaerobic sediment of the deep sea. Moreover, the whole cell protein was increased in the lignin supplemented group, which indicated that the novel isolated strain has a strong ability to decompose refractory polysaccharide. Strain NS-1 could produce a large number of different fermentation products including hydrogen, alcohol, acetate, and butyrate, and the amount of these small molecular substrates varied accordingly to the substrate categories (Figure 4). Notably, alginate could significantly increase the production of hydrogen and acetate (Figures 4A,C) without a significant increase in growth (Figure 2). Therefore, acting as an active degrader of organic matter in anaerobic sediments, which are inhabited by large amounts of autotrophic-heterotrophic microorganisms, *I. fonsfrigidae* NS-1 is proposed to promote the elements and nutrient cycling in the dark biosphere by providing a variety of small molecular organic nutrients.

Comparative genomic results indicated that strain NS-1 and *Halocella* sp. SP3-1 probably belong to the same species (Supplementary Figure 6). The most obvious difference between these two strains is the presence of a variety of genes associated with BMC constitution and ethanolamine and propanediol metabolism in the genome of strain NS-1 but the absence in the genome of *Halocella* sp. SP3-1 (Figure 6A). BMCs are organelles that encapsulate functionally linked enzymes within a proteinaceous shell. They play crucial roles in carbon dioxide fixation in autotrophs (Rae et al., 2013) and the catabolism of organic substrates in heterotrophs (Kofoid et al., 1999; Erbilgin et al., 2014). They were reported to be involved in the degradation of a number of plant and algal cell wall derived sugars, and L-fucose and L-rhamnose could promote the formation of BMCs in *Planctomyces limnophilus* (Erbilgin et al., 2014). It was also reported that BMCs were responsible for the degradation of plant saccharides (Erbilgin et al., 2014). Correspondingly, the expression levels of most genes in the BMC related gene cluster were dramatically up-regulated when supplementing CMC, lignin, or glucose in the medium (Figure 6B), strongly suggesting that ethanolamine and propanediol metabolisms were affected by the metabolized products of different carbohydrates such as ethanol and propanol (Figure 6B). The existence of BMC in strain NS-1 may contribute to metabolic versatility, and provide a competitive advantage in specific environmental niches.

Taken together, based on the isolate *I. fonsfrigidae* NS-1, we explored its physiology, genomic traits, phylogenetics, and metabolisms in detail, through bioinformatics, biochemical and transcriptomic methods, and proposed a model describing its central metabolic pathways (Supplementary Figure 4). The metabolisms of diverse carbohydrates (such as cellulose, lignin) are crucial for the complete metabolic network of this novel strain and closely link to other important metabolic processes like ethanolamine and propanediol, which are conducted in the BMC. Besides the contribution of diverse and enormous small molecular organic matter to the surrounding environment, it is also proposed that it might be involved in elements cycling such as sulfur and metal ions, which need to be investigated further in the future.

## Data Availability Statement

The NCBI GenBank accession numbers for the 16S rRNA gene sequence and whole genome sequence (WGS) of strain NS-1 are MK418264.1 and CP046640, respectively. The access number for the original transcriptome data is PRJNA666065 in the NCBI GenBank.

## Author Contributions

JZ and CS conceived and designed the study. JZ performed the most of the experiments. YZ and FL measured the anaerobic products of NS-1. RL collected the samples. JZ, RC, and CS analyzed the data. JZ wrote the manuscript. CS revised the manuscript. All authors read and approved the final manuscript.

## Conflict of Interest

The authors declare that the research was conducted in the absence of any commercial or financial relationships that could be construed as a potential conflict of interest.

## Publisher’s Note

All claims expressed in this article are solely those of the authors and do not necessarily represent those of their affiliated organizations, or those of the publisher, the editors and the reviewers. Any product that may be evaluated in this article, or claim that may be made by its manufacturer, is not guaranteed or endorsed by the publisher.
