# Supplementary material for: Iocasia fonsfrigidae NS-1 gen. nov., sp. nov., a Novel Deep-Sea Bacterium Possessing Diverse Carbohydrate Metabolic Pathways
Source: Front Microbiol. 2021 Nov 24;12:725159. doi: 10.3389/fmicb.2021.725159 (PMC8652127; doi:10.3389/fmicb.2021.725159)
Supplement: Supplementary file 3 [file Table_3.DOCX]

**Supplementary information**

***Iocasia fonsfrigidae* NS-1 gen. nov., sp. nov., a novel deep-sea bacterium possessing diverse carbohydrate metabolic pathways**

Jing Zhang^1,2,3,4,6^, Yuechao Zhang^5^, Rui Liu^1,2,4^, Ruining Cai^1,2,3,4^, Fanghua Liu^5^, Chaomin Sun^1,2,4 *^

^1^CAS Key Laboratory of Experimental Marine Biology & Center of Deep Sea Research, Institute of Oceanology, Chinese Academy of Sciences, Qingdao, China

^2^Laboratory for Marine Biology and Biotechnology, Qingdao National Laboratory for Marine Science and Technology, Qingdao, China

^3^College of Earth Science, University of Chinese Academy of Sciences, Beijing,

China

^4^Center of Ocean Mega-Science, Chinese Academy of Sciences, Qingdao, China

^5^Key Laboratory of Coastal Biology and Biological Resources Utilization, Yantai

Institute of Coastal Zone Research, Chinese Academy of Sciences, Yantai, China

^6^ School of Life Sciences, Hebei University


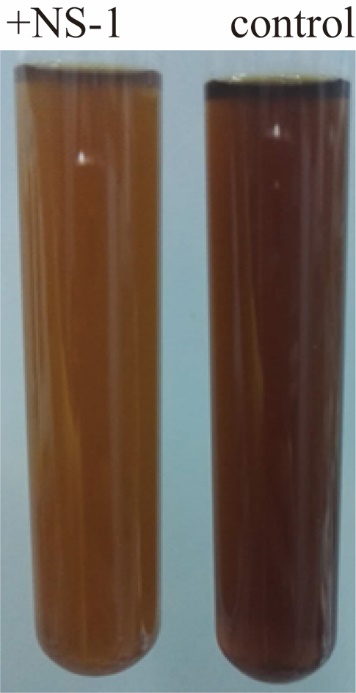


**Figure S1** Detection of the lignin degradation by strain NS-1. The color of medium containing lignin without inoculation is dark, however, it became a bit clearer when incubated with strain NS-1, indicated the consumption of lignin by strain NS-1.


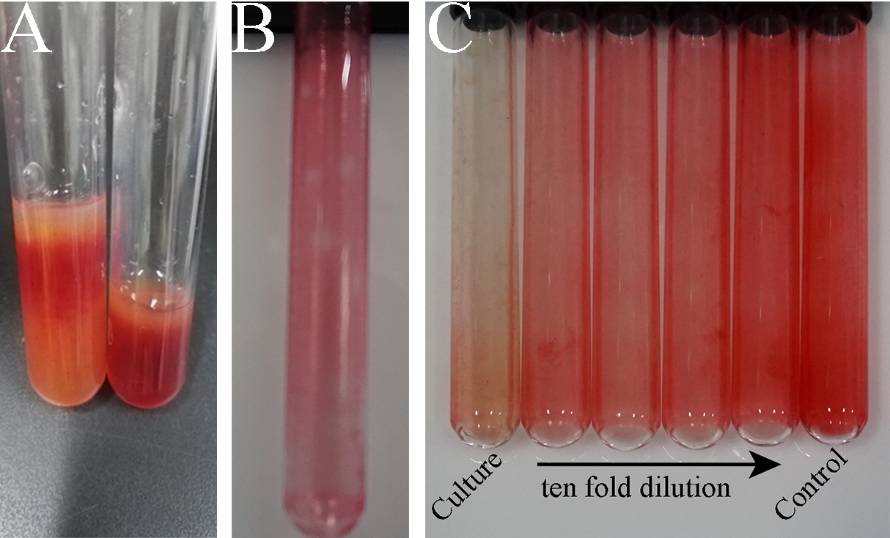


**Figure S2.** Detection of the CMC degradation capability of strain NS-1 by measuring the medium consumption (A) and observation of the transparent zones (B, C). In panel C, the color of Congo red became darker along with the decrease of the amount of strain NS-1 by series of dilution, indicating the transparent zones were corresponding to the CMC degradation areas.


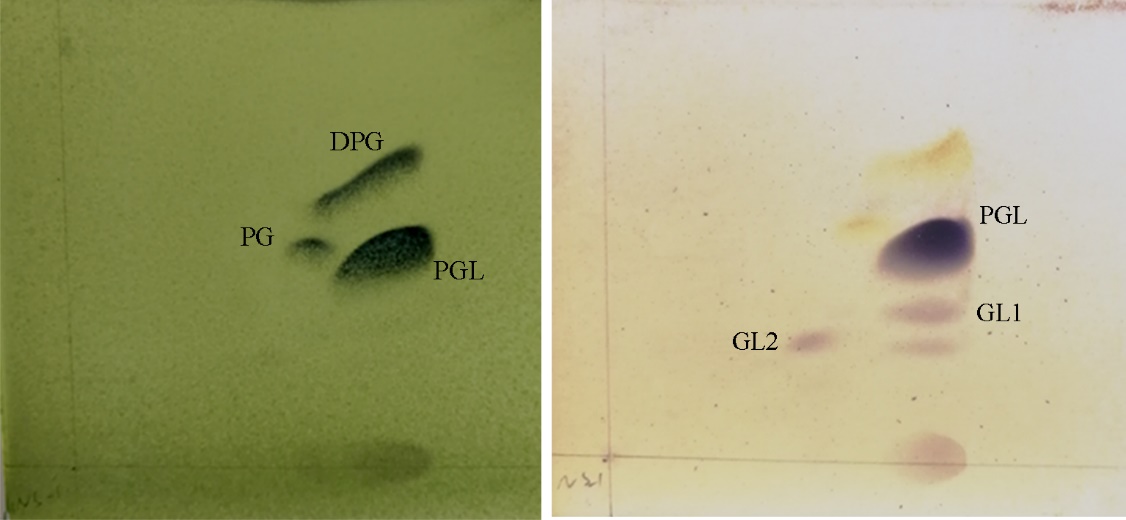


**Figure S3** Composition of polar lipids in strain NS-1. DPG, diphosphatidylglycerol, PG, phosphatidylglycerol, PGL, unidentified phosphoglycolipids, GL1 and GL2, two kinds of unidentified glycolipids.


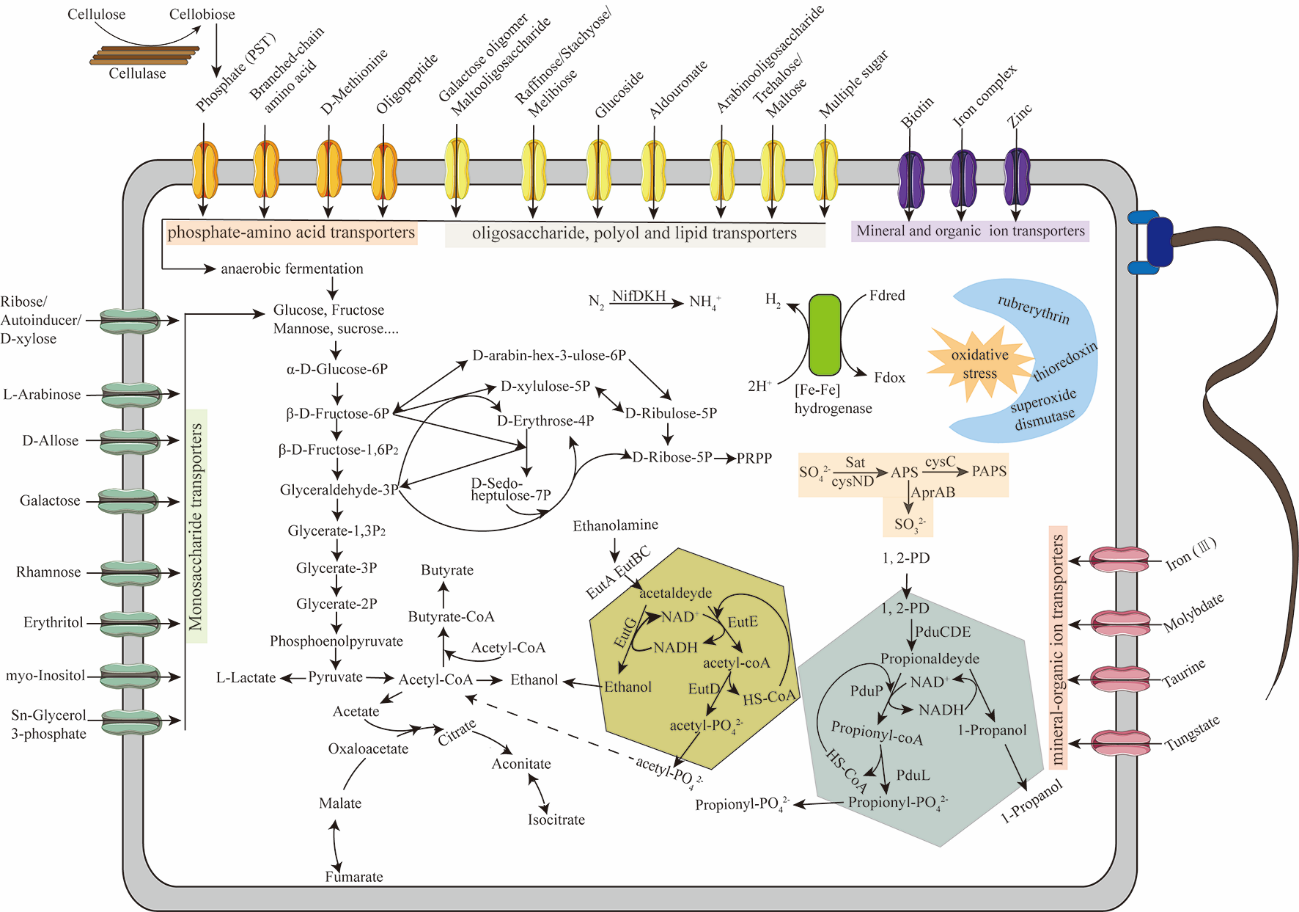


**Figure S4** Reconstructed metabolic pathways of *I. fonsfrigidae* NS-1 based on genomic analysis. The solid lines indicate the metabolic pathway associated genes are present in the genome, while the dashed lines indicate the metabolic pathway associated genes are absent in the genome. In this diagram: EutA, EutBC, EutE and EutG indicate ethanolamine utilization protein; 1, 2-PD indicates 1,2-propanediol; PduCDE, PduP and PduL indicate propanediol utilization proteins. Sat: sulfate adenylyltransferase, cysND: bifunctional enzyme CysN/CysC, AprAB: adenylylsulfate reductase.


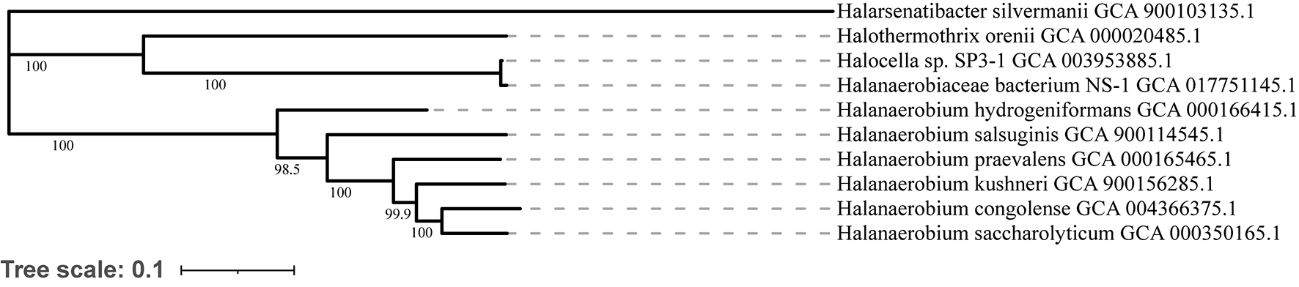


**Figure S5** Maximum likelihood phylogenetic tree of the genomes of 10 cultured strains in the family Halanaerobiaceae was reconstructed using concatenated alignment of 37 single-copy genes by Phylosift (v1.0.1) and W-IQ-TREE with GTR+F+I+G4 model. The aligned sequences were trimmed by trimaL (v1.2) with gappyout function. The final tree was completed by itol (V6).


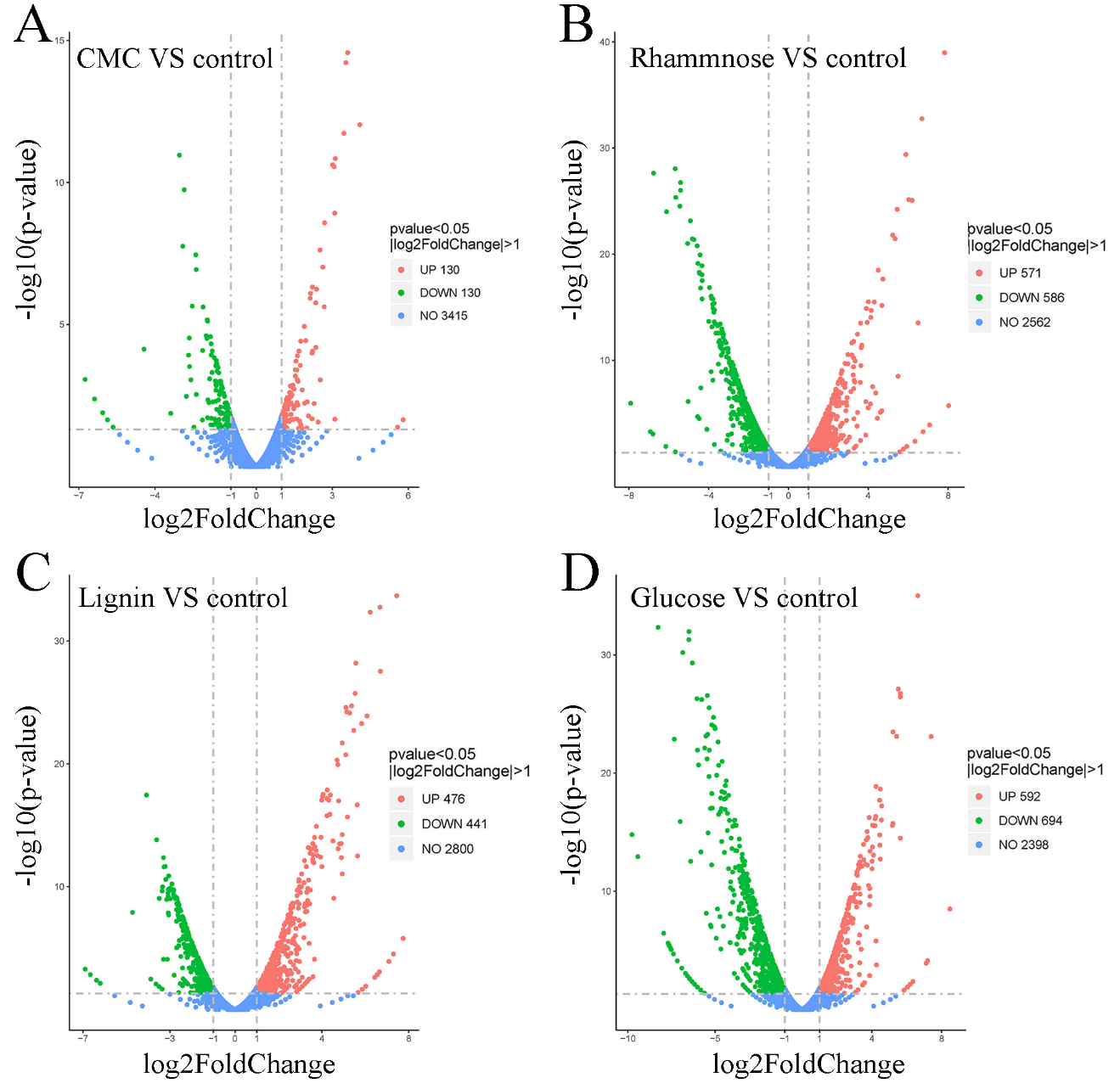


**Figure S6.** Volcano plot displaying differentially expressed genes in strain NS-1 cultured with different carbohydrates (CMC, rhamnose, lignin, glucose) when compared to the control group.


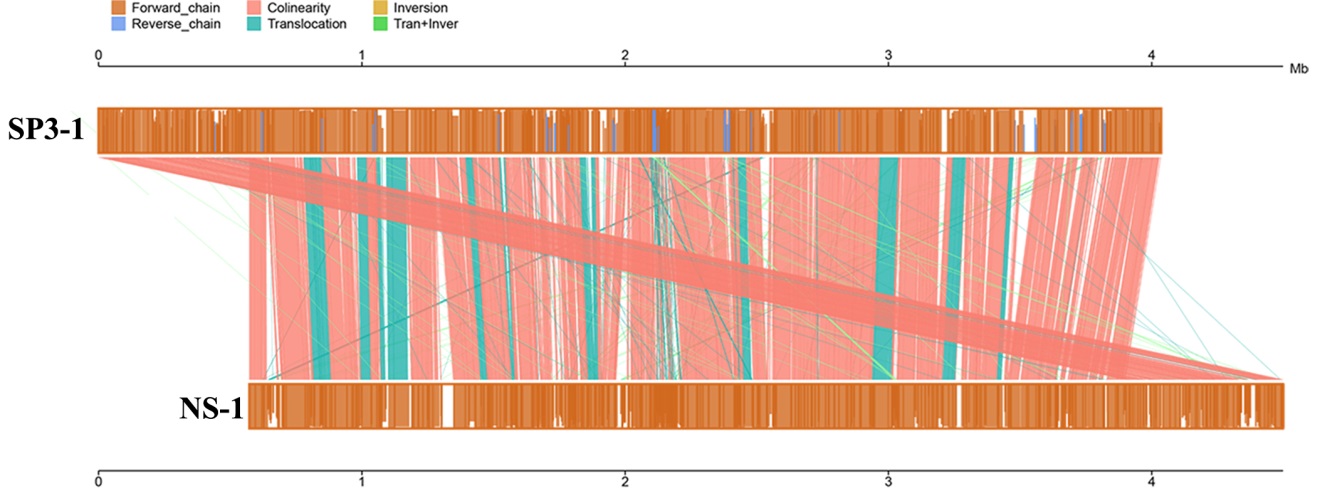


**Figure S7.** Genomic comparison between genomes of strain NS-1 and *Halocella* sp. SP3-1.

**Table S4** ANI values between strain NS-1 and type strains in the family Halanaerobiaceae

| strain | ANI value (%) | |
| --- | --- | --- |
| Halanaerobiaceae bacterium NS-1 | | 100 |
| Halanaerobium praevalens | | 74.8473 |
| Halothermothrix orenii | | 75.1698 |
| Halarsenatibacter silvermanii | | NA |

NA means the value was lower than 70%

**Table S5** Characteristics of the new genus *Iocasia* and other genera in the family Halanaerobiaceae

| genus | *Iocasia* | *Halocella* [1] | *Halothermothrix* [2] | *Halarsenatibacter* [3] | *Haloanaerobium* [4] |
| --- | --- | --- | --- | --- | --- |
| Gram | Negative | Negative | Negative | Negative | Negative |
| cell size | 0.2-0.3 ×6-10 μm | N | 0.4-0.6 × 10-20 μm | 0.5 × 3 μm | 0.9-1.1× 2-2.6μm |
| morphology | long rods | N | rods | N | rods |
| motility | + | N | + | + | - |
| needs of oxygen | obligately anaerobic | obligately anaerobic | strictly anaerobic | strictly anaerobic | N |
| needs of sodium chloride | moderately halophilic | moderately halophilic | obligately haophilic | extreme halophile | N |
| NaCl range | 1.25-15% | N | N | 20-35% | 2-30% |
| NaCl optimum | 2.5-7.5% | N | N | 35% | 13% |
| pH range | 6.5-8 | N | N | 8.7-9.8 | 6.0-9.0 |
| pH optimum | 7 | N | N | 9.4 | 7.0-7.4 |
| temperature range | 20-45 ^o^C | N | N | 28-55 ^o^C | 5-50 |
| temperature optimum | 37 ^o^C | N | N | 44 ^o^C | 37 ^o^C |
|  | 14:0 15:0iso 15:0 anteiso | 14:0 16:0 15:0anteiso | N | 15:0iso 18:0 17:0iso 16:0 | 14:0 16:0 16:1 |
| G+C content of DNA (mol %) | 35.1-35.7% | 29 | 39.6 | 45.2 | 27 |

^*^ “+” indicates the strain has that capability; “-” indicates the strain lacks that capability.

^#^ N indicates not provided.

Related reference cited in this table, [1] (Simankova *et al.* 1993), [2] (Cayol *et al.* 1994), [3] (Blum *et al.* 2009), and [4] (Zeikus *et al.* 1983).

**Reference**

Simankova M., Chernych N., Osipov G.*, et al.* 1993. *Halocella cellulolytica* gen. nov., sp. nov., a new obligately anaerobic, halophilic, cellulolytic bacterium. Syst Appl Microbiol **16**: 385-389. doi:10.1016/S0723-2020(11)80270-5

Cayol J.-L., Ollivier B., Patel B.*, et al.* 1994. Isolation and characterization of *Halothermothrix orenii* gen. nov., sp. nov., a halophilic, thermophilic, fermentative, strictly anaerobic bacterium. Int J Syst Evol Microbiol **44**: 534-540. doi:10.1099/00207713-44-3-534

Blum J. S., Han S., Lanoil B.*, et al.* 2009. Ecophysiology of “*Halarsenatibacter silvermanii*” strain SLAS-1T, gen. nov., sp. nov., a facultative chemoautotrophic arsenate respirer from salt-saturated Searles Lake, California. Appl Environ Microbiol **75**: 1950-1960. doi:10.1128/AEM.02614-08

Zeikus J., Hegge P., Thompson T.*, et al.* 1983. Isolation and description of *Haloanaerobium praevalens* gen. nov. and sp. nov., an obligately anaerobic halophile common to Great Salt Lake sediments. Curr Microbiol **9**: 225-233. doi:10.1007/BF01567586
